# The Many Unknowns Concerning the Bioenergetics of Exhaustion and Senescence during Chronic Viral Infection

**DOI:** 10.3389/fimmu.2014.00468

**Published:** 2014-09-25

**Authors:** Anna Schurich, Sian M. Henson

**Affiliations:** ^1^Division of Infection and Immunity, University College London, London, UK

**Keywords:** exhaustion, senescence, chronic viral infection, bioenergetics, metabolism

## Abstract

The immune system cannot be continuously reactivated throughout the lifetime of an organism; there is a finite point at which repeated antigenic challenge leads to the loss of lymphocyte function or the cells themselves. Antigen-specific T cells can be compromised in two ways through the distinct processes of replicative senescence and exhaustion. Senescence is initiated by a DNA damage response whereas exhaustion triggers inhibitory receptors to dampen the immune response. These two distinct pathways not only differ in their initiation but also growing evidence suggests that their biogenergetics is also different. Here, we review recent findings uncovering the metabolism of these unique states.

## Metabolism of the Immune Response to Viral Infection

During viral infections CD8^+^ T cells undergo clonal expansion and produce effector molecules. In order to meet this bioenergetic demand, the cells switch their metabolism from a mitochondrial-dependent oxidative and fatty acid metabolism (1, 2), toward glycolysis and glutamine oxidation (3, 4), even in the presence of sufficient oxygen, a process termed aerobic glycolysis. Activated T cells use this metabolism, although it is less energy efficient, as it allows for the increase in biomass through the biosynthesis of fatty acids and nucleotides (3). Cytokines and co-stimulatory signals via CD28 help promote the metabolic switch (5, 6). This acute phase of the viral response is then followed by a contraction phase, characterized by a decrease in mitochondrial membrane potential and high levels of reactive oxygen species (ROS), which triggers the majority of the antigen-specific effector cells to be cleared by apoptosis (7). The remaining antigen-specific cells differentiate into long-lived memory cells that protect against recurrent infection. The metabolism also changes within the memory population; memory T cells revert back to fatty acid oxidation and preferentially use the TCA cycle to fuel oxidative phosphorylation (OXPHOS) (2). These changes appear to be governed by IL-15, an important cytokine for CD8^+^ memory T cells, as it promotes mitochondrial biogenesis and regulates the mitochondrial spare respiratory capacity, the extra capacity available in cells to produce energy (2, 8).

Although numerous infections are successfully cleared by the acute immune response, certain viral infections are not resolved and result in chronicity. The function of virus-specific T cells during chronic infections is often characterized by varying degrees of impairment, leading to defects in the ability of the host to eliminate the pathogen. Depending on the antigenic load generated by the infectious agent the impaired memory formation can result in T cells becoming either senescent or exhausted (9). Thus, immune senescence arises as a consequence of low-grade antigenic stimulation with the resulting inflammation determining the rate of senescence, as seen with CMV or EBV infection and not age *per se* (10, 11). The quantity of the lifelong antigenic load and the resulting inflammation determines the rate of immune senescence. Whereas a high-antigen load, caused by HIV, HCV, and HBV infection, leads to the formation of exhausted T cells (12). In this present review, we will discuss the current understanding of the metabolic requirements of antigen-specific CD8^+^ T cells in chronic infections.

## Senescence and Exhaustion are Distinct Processes

**Figure 1:**
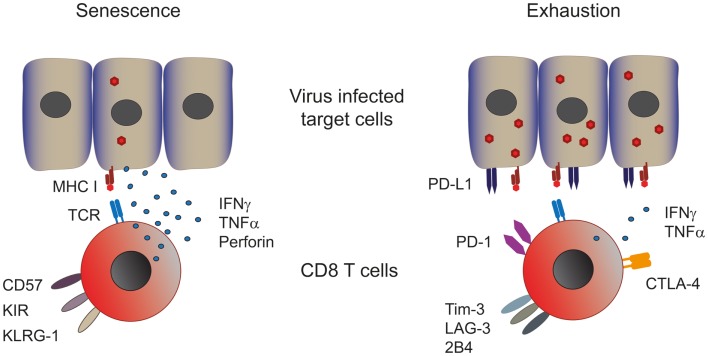
**Phenotype and function of senescent and exhausted T cells**. Senescent T cells are present in chronic viral infections with a low viral and antigenic load. Senescent T cells express CD57, the late activation marker killer-cell lectin-like receptor subfamily G member 1 (KLRG-1) and killer-cell immune globulin-like receptors (KIR) and are capable of producing significant amounts of effector cytokines such as perforin, IFN-γ, and TNF-α (left panel). Exhausted CD8 T cells are found in persistent infections with a high viral and antigenic load. These cells express various co-inhibitory receptors such as PD-1, CTLA-4, Tim-3, LAG-3, and 2B4, which dampen their effector response consequently exhausted T cells produce low amounts of effector cytokines (right panel). Both T cell types show impaired proliferative capacities.

As introduced above two different cellular processes can lead to T cell dysfunction, namely, senescence and exhaustion (Figure 1). Numerous mechanisms have been proposed to cause cellular senescence, including repeated cell division, telomere shortening, and damage by ROS (13, 14). The ensuing DNA damage triggers the recruitment of a complex of proteins that are involved in DNA repair, which is commonly referred to as the DNA damage response (DDR) that inhibits cell cycling until the DNA is repaired (13, 15, 16). Senescence manifests itself in T cells as the loss of the co-stimulatory molecule CD28 and the acquisition of innate markers such as killer-cell lectin-like receptor G1 (KLRG-1), while senescent T cells lose proliferative capacity they retain their cytotoxic activity and secretion of TNFα and IFNγ (17–19).

Continuous T cell stimulation in the setting of a high-antigenic load induces a state termed T cell exhaustion, characterized by the loss of effector functions in a hierarchical manner (20). IL-2 production and proliferation are the first functions to be lost, followed by TNFα production and cytotoxic activity. At late stages of exhaustion, IFNγ production is eventually compromised. When the antigen persists long-term at high-levels exhausted T cells are ultimately removed by apoptosis. So in contrast to senescent T cells, which can produce high amounts of effector cytokines, exhausted T cells do not. Both groups have low-proliferative potential in common. Furthermore, functional exhaustion is accompanied by a marked change in T cell phenotype. Expression and maintenance of the co-inhibitory receptors programed cell death-1 (PD-1) at high levels is a hallmark of exhausted T cells, concomitantly other inhibitory receptors such as cytotoxic T lymphocyte antigen-4 (CTLA-4), lymphocyte activation gene-3 (LAG-3), T cell immunoglobulin and mucine domain containing molecule-3 (Tim-3), CD160 (21), and the natural killer-cell receptor 2B4 can also be significantly increased (12). T cell dysfunction in exhaustion is, at least in part, mediated by these inhibitory receptors, since multiple studies have demonstrated that their blockade results in functional recovery of exhausted T cells, examples include PD-1 blockade in HIV, HBV, and HCV (22–25) and CTLA-4 and Tim-3 blockade in HBV (26, 27).

Both senescent and exhausted T cells show a differentiation profile distinct from memory or effector cells. Senescent human CD8^+^ T cells express high levels of T-bet but only a moderate amount of EOMES (28). Exhausted T cells express high levels of the transcriptional repressor Blimp-1, responsible for the increased expression in co-inhibitory receptors (29). NFATc1, is also increased and surprisingly is associated with poor cytokine expression (12), while T-bet expression in exhausted T cells seems to be important in supporting their persistence and sustenance of any residual functionality (12). However, exhausted T cells do help to control viral levels both in chronically infected patients and shown experimentally in elegant T cell transfer studies in murine chronic lymphocytic choriomeningitis virus (LCMV) (30). It is tempting to speculate that both T cell senescence and exhaustion are mechanisms by which viral infections are kept under control in order to avoid extensive on going immune damage.

## Metabolism in Senescent and Exhausted CD8 T Cells

**Figure 2:**
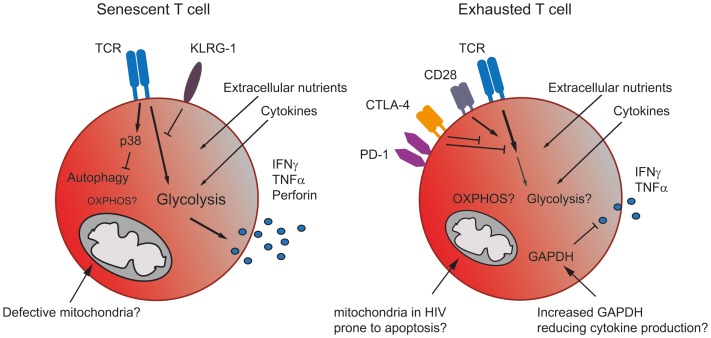
**The metabolism in senescent and exhausted T cells**. Senescent T cells rely more on glycolysis than mitochondrial respiration due to the accumulation of giant non-functional mitochondria in these terminally differentiated cells. The inhibitory receptor KLRG-1 prevents signaling through the TCR, while the activation of p38 blocks autophagy, which senescent T cells use as an energy source. Nonetheless, senescent T cells generate sufficient energy to produce significant amounts of effector cytokine (left). Exhausted T cells express co-inhibitory receptors, which interfere with TCR and co-stimulatory signaling, thereby likely blocking any increase in metabolic activity. Cells may also be prone to mitochondrial induced apoptosis. Additionally, an increase in GAPDH, caused for example by lower levels of glycolysis, might also dampen effector cytokine production (right).

Senescent and exhausted T cells display a metabolic phenotype distinct from memory cells but also, due to their differing functionality, from each other (Figure 2). Senescent human CD8^+^ T cells isolated from healthy donors stimulated through the TCR have been shown to preferentially utilize glycolysis and also exhibit mitochondrial dysfunction and impaired mitochondrial biogenesis, which may explain their dependence on glycolysis for energy (17). The ability of a T cell to undergo mitochondrial biogenesis leads to an increased capacity of the cell to respond to metabolic stress, a characteristic termed spare respiratory capacity (2). It has been demonstrated that, unlike other memory subsets senescent CD8^+^ T cells have a substantially reduced spare respiratory capacity making them energetically unstable (17).

Information regarding the metabolism of exhausted T cells is limited and, to date, restricted to mouse models where alterations in metabolism have been reported. During acute infection with the LCMV Armstrong clone, virus-specific CD8 T memory cells develop. In contrast, during chronic infection with the LCMV clone 13, repetitive antigen-stimulation leads to the loss of memory cells. Virus-specific cells detectable in chronically infected animals were either still naïve or functionally exhausted (31). Wherry et al. have demonstrated using exhausted CD8^+^ T cells following chronic infection with LCMV that genes involved in glycolysis and oxidative metabolism were significantly altered compared to effector and memory CD8 T cells (32), suggesting that exhaustion is a distinct state.

Information regarding the metabolic requirements of HIV is hampered by the toxicity of many of the antiretrovirals (33). However, removal of mitochondrial content from a cell line leads to the attenuation of HIV infection (34). Furthermore, cells isolated from HIV^+^ patients may up-regulate OXPHOS owing to an increased mitochondrial mass (33). However, the methodology used in this study cannot distinguish whether the observed increase is due to an increased number of functional mitochondria or the appearance of giant non-functional mitochondria (35). From the limited available data, it would appear that T cells utilize OXPHOS during infections with both LCMV and HIV; however, it remains to be investigated whether T cells specific to different pathogens being primed in different locations in the body establish distinct metabolic profiles. Furthermore, when examining human infections the length of time post-infection may also be a deciding factor in the metabolic fate of T cells, as repeated turnover may lead to mitochondrial dysfunction and a switch toward extra-mitochondrial metabolism (36).

One hallmark of immune senescence is the accumulation of late-differentiated effector T cells characterized by the loss of CD28 expression (9, 37, 38). Exhausted T cells have been described to also display an effector memory T cell phenotype (39). Signaling through CD28 has been demonstrated to increase glycolysis and therefore effector function in CD4^+^ T cells. Both PD-1 and importantly CTLA-4, a direct competitor of CD28, interfere with CD28 signaling (40, 41), their increased expression on exhausted T cells is therefore likely to dampen down a metabolism suitable to sustain effector functions. While the CD28 co-stimulation pathway is considered to be important for T cell activation, alternative co-stimulatory pathways belonging to the TNF/TNF receptor (TNFR) family have been described. Signaling through CD137:CD137L (4-1BB) has been demonstrated to promote proliferation of CD8^+^CD28^−^ T cells and is an important co-stimulator for human anti-viral CD8 T cells, as a CD137 agonist given alongside PD-L1 blockade resulted in an enhanced and stable expansion of LCMV-specific CD8^+^ T cells (42).

Furthermore, while not directly examining exhaustion Chang et al. have shown using mice infected with *Listeria* that forcing CD4^+^ T cells to use OXPHOS induced elevated PD-1 expression, a loss of proliferation and defective IFN-γ production (43). However, what is hard to reconcile from this study is quiescent memory T cells, which utilize OXPHOS do not express PD-1 (2, 32), therefore, the elevated levels of PD-1 observed in this scenario may be controlling activation. Additionally, *in vivo* it is questionable whether T cells will find themselves in a situation where they are forced to utilize OXPHOS. T cell exhaustion and senescence is pathogen specific with generally little impact on the overall response to other infections and T cells specific to various different viruses can accumulate in the same location (44). Finally, memory, effector, and dysfunctional T cells can share the same environment, while displaying distinct metabolism, suggesting that a regulation through predominantly T cell intrinsic rather than extrinsic factors. So the question of what drives the metabolic phenotype of dysfunctional cells remains.

## Influence of Metabolic Regulators on Senescent and Exhausted CD8 T Cells

Metabolism can be controlled by many different immunological and metabolic signals [reviewed in Ref. (45)], how chronic infections influence these metabolic checkpoints remains to be determined (Figure 2). Cytokines have been shown to regulate metabolism, indeed IL-2 can induce the expression of glucose transporter 1 (glut1) and enhance glycolysis (46). Both exhausted and senescent T cells show no or reduced production of IL-2. In line with this human senescent CD8^+^ T cells show a reduced expression of glut1 (17). The glut1 level on exhausted T cells has not been examined. The homeostatic cytokines IL-7 and IL-15 also regulate metabolism, IL-7 promotes glycolysis in T cells (47, 48) and IL-15 has been demonstrated to regulate oxidative metabolism by enhancing mitochondrial biogenesis in memory CD8^+^ T cells but not effectors (2). Type 1 IFN, a prevalent cytokine during viral infections induces the production of IL-15 (49), which may also add to the oxidative switch during viral infections. Additionally, type 1 IFN has also been demonstrated to modulate lipid metabolism (50), while lipid metabolism is crucial for memory T cells, a role for this cytokine in the control of metabolism during senescence and exhaustion, where effector T cells dominate is unclear.

The distinct nature of the metabolic changes seen during chronic infection suggests additional regulatory steps in the metabolic reprograming of T cells. The transcription factor mammalian target of rapamycin (mTOR), as well as being a critical regulator of CD8^+^ memory formation (51), is a key molecule sensing intracellular amino acids and ATP (52), and regulates fatty acid metabolism in memory T cells (1). The pro-inflammatory cytokine IL-12 increases mTOR expression in antigen-stimulated CD8^+^ T cells, promoting CD8^+^ T effector differentiation and metabolism. Blockade of mTOR by rapamycin led to inhibition of IL-12 induced expression of the transcription factor T-bet and skewed the CD8 response toward eomesodermin dependent memory formation (53). T-bet is important in maintaining the limited effector capacity of T cells in chronic infections (54) and IL-12 enhances functionality of exhausted T cells in chronic HBV by increasing T-bet (55). It has recently been shown that senescent CD8^+^ T cells display very little mTOR activity and predominantly use mTOR-independent pathways to control their metabolic requirements (17). This is in line with the concept that the transition to a memory phenotype is associated with a metabolic switch from anabolism to catabolism (41, 52) via the inhibition of mTOR (51). This limited mTOR activity also corresponds to the lower level of glut 1 observed on senescent T cells compared to other memory populations (17).

This review has focused on the changes occurring to glycolysis and oxidative metabolism during chronic infections. However, alternative energy sources, such as the β-oxidation of fatty acids or autophagy may be utilized differentially by senescent and exhausted T cells. A recent article by the Pearce group has demonstrated that the fatty acids required by memory T cells are produced *de novo* via the non-classical lysosomal acid lipase (LAL) pathway to mobilize fatty acids for β-oxidation (56). This study uses the mouse OVA system together with cytokines to generate effector and memory cells, but the observed pathways have not yet been shown to occur in humans. Furthermore, while both senescent and exhausted T cells are not highly proliferative, like memory cells, they do make cytokine and senescent T cells also express high levels of effector molecules necessitating a higher catabolic demand. Macroautophagy (autophagy) is another alternate nutrient source when extracellular nutrient uptake is insufficient to meet the cellular energy demands (57). The lysosomal digestion of organelles and other materials by autophagy can generate the required metabolic precursors for metabolism (57). Furthermore, mouse models have shown autophagy to support glycolysis and autophagy competence is required for cells to proliferate and expand (58). Senescent human CD8^+^ T cells display low-autophagic activity (17, 59), which was regulated via p38 MAPK independently of mTOR (17). However, the role of both fatty acid metabolism and autophagy during T cell exhaustion remains to be determined.

## Summary

Although the interest in metabolism has grown significantly and our understanding of the specific requirements of T cells with it, there is still very little understanding of how T cells fuel their energy demand during chronic viral infections. We have highlighted here the distinct phenotypes and functions of exhausted and senescent CD8 T cells and have outlined the current knowledge of their metabolic requirements. However, many questions remain open: it remains to be seen, how much influence the environment the T cell is located in at any given stage during the infection has. Are metabolic phenotypes at least partially imprinted during T cell priming in the acute phase of the infection or do they only develop over time? How big is the influence of the antigen presenting cell and the local environment during priming? For example, HBV/HCV-specific T cells are likely to encounter their cognate antigen for the first time in the liver and be primed there, whereas EBV-specific T cells might be primed in the tonsils. The liver milieu is naturally high in suppressive cytokines (60, 61), which control and dampen T cell activation and might influence the metabolic pathway used. Furthermore, it is likely that the immune response differs when taking place in a hypoxic environment compared to one with higher oxygen supply. Finally, the infecting virus itself will influence the T cell response by manipulating cytokine, chemokine, and co-stimulatory or co-inhibitory receptor expression.

These questions have so far not been taken into account when investigating chronic infections, since most studies have utilized only the murine LCMV model to study T cell dysfunction. The limitation being that, specific changes in T cell phenotype and function caused by distinct infections have not been addressed. Ultimately, the aim will have to be to better understand human T cell responses in order to allow the development of novel immunotherapies. Albeit the difficulties of establishing reliable *in vitro* models and understanding the mechanisms governing human anti-viral immunity, studies making use of human samples need to be further encouraged.

## Conflict of Interest Statement

The authors declare that the research was conducted in the absence of any commercial or financial relationships that could be construed as a potential conflict of interest.
